# Monocyte Differentiation on Atomic Layer-Deposited (ALD) Hydroxyapatite Coating on Titanium Substrate

**DOI:** 10.3390/molecules28083611

**Published:** 2023-04-21

**Authors:** Elina Kylmäoja, Faleh Abushahba, Jani Holopainen, Mikko Ritala, Juha Tuukkanen

**Affiliations:** 1 Department of Anatomy and Cell Biology, Institute of Cancer Research and Translational Medicine, Medical Research Center, University of Oulu, P.O. Box 5000, 90014 Oulu, Finland; 2 Department of Prosthetic Dentistry and Stomatognathic Physiology, Institute of Dentistry, University of Turku, 20520 Turku, Finland; 3 Department of Chemistry, University of Helsinki, P.O. Box 55, 00014 Helsinki, Finland

**Keywords:** atomic layer deposition, hydroxyapatite, titanium, osteoclast, resorption

## Abstract

Hydroxyapatite (HA; Ca_10_(PO_4_)_6_(OH)_2_) coating of bone implants has many beneficial properties as it improves osseointegration and eventually becomes degraded and replaced with new bone. We prepared HA coating on a titanium substrate with atomic layer deposition (ALD) and compared monocyte differentiation and material resorption between ALD-HA and bone. After stimulation with macrophage colony-stimulating factor (M-CSF) and receptor activator of nuclear factor kappa-B ligand (RANKL), human peripheral blood monocytes differentiated into resorbing osteoclasts on bovine bone, but non-resorbing foreign body cells were observed on ALD-HA. The analysis of the topography of ALD-HA and bone showed no differences in wettability (water contact angle on ALD-HA 86.2° vs. 86.7° on the bone), but the surface roughness of ALD-HA (Ra 0.713 µm) was significantly lower compared to bone (Ra 2.30 µm). The cellular reaction observed on ALD-HA might be a consequence of the topographical properties of the coating. The absence of resorptive osteoclasts on ALD-HA might indicate inhibition of their differentiation or the need to modify the coating to induce osteoclast differentiation.

## 1. Introduction

Besides the ancient efforts to replace missing teeth and repair injured tissues and the progression to the first documented use of a metal implant in 1565, it was not until the beginning of the 20th century that implants became a vastly used treatment for tissue replacement. During the last century, several types of metals, polymers, and ceramics with various coatings and surface modifications have been developed in an attempt to meet the increasing demand for implantable devices [1].

Orthopedic implants are one of the most common synthetic devices for tissue engineering [2]. The properties of an ideal bone biomaterial have been described by Bose et al. [3], including biocompatibility, suitable mechanical properties, porosity, and resorbability. Concerning biocompatibility in bone tissue, the terms osteoconductive and osteoinductive have been used to describe the necessary bone cell adhesion, proliferation, and bone ingrowth, as well as the promotion of new bone formation by immature cell differentiation and osteogenesis, respectively [3,4]. Together, osteoconduction and osteoinduction lead to osseointegration, described as a direct contact between living bone and the implant [5]. Failed osseointegration is the main reason for implant failure, often leading to implant loosening and the need for revision surgery. This causes a burden for the patients and the whole healthcare system and increases the total costs of orthopedic treatment [6,7,8,9].

The natural bone and teeth mineral hydroxyapatite (HA; Ca_10_(PO_4_)_6_(OH)_2_) has been utilized in bone implant coating since it can improve osseointegration [10,11,12,13,14,15]. The stable fixation of the implant is based on the direct growth of bone tissue into the HA coating [14,16,17], the subsequent degradation of HA, and the final replacement by new bone [18,19,20,21]. The bone degradation phase is conducted by the special multinuclear bone cells, osteoclasts, which are also active during normal bone homeostasis. The complex bone microarchitecture is sustained and renewed by the cooperation of the bone-resorbing osteoclasts, bone-forming osteoblasts, and mechanosensing osteocytes [22]. This activity, termed remodeling, also occurs at the bone-HA interface and is a crucial process for implant fixation into bone tissue [18,20,23,24,25,26,27,28,29].

Upon implantation of a foreign material in the body, a foreign-body reaction begins around the implant. The reaction includes the migration of mononuclear cells at the implantation site and their differentiation into multinuclear macrophages, also termed foreign body giant cells (FBGC), which try to phagocytose the foreign material or degrade it by secreting reactive oxygen species (ROS) and matrix metalloproteases (MMPs) [30,31]. FBGCs cells bear a resemblance to the bone-resorbing osteoclasts, except that the FBGCs are larger and cannot resorb bone tissue [31,32,33,34]. However, the foreign body cells can produce cytokines and other mediators promoting osteoclastogenesis [35,36,37], or they can directly differentiate into functional osteoclasts [38,39]. This leads to increased bone resorption and osteolysis around the implant, a common reason for implant loosening.

Considering HA, several studies have shown that FBGCs are induced by HA-particle stimulation [40,41,42,43] and that the HA-induced FBGCs from mouse subcutaneous tissue can differentiate into osteoclasts [44]. Furthermore, although FBGCs do not resorb bone, they have been shown to dissolve HA-like calcium phosphate coating [32].

Osteoclasts can resorb HA [18,19,21,25,32,45,46], but there are also contrary results showing no resorption on pure HA [47,48]. The contradictory results can be explained by the studies, which suggested that the surface topography of the HA coating has major consequences on osteoclast differentiation and function [49,50,51,52,53,54,55] and that increasing the HA content in calcium phosphate materials inhibits osteoclastogenesis [56]. In fact, the implant chemistry and topography affect the whole initial foreign body reaction and the complex cellular interactions thereafter [57,58,59,60].

A variety of methods have been utilized to synthesize calcium phosphate coatings. Examples include sol-gel, plasma spraying, laser ablation, electrophoretic deposition, and sputter coating, but all these methods have been found to have challenges concerning the phase or the stability of the coatings. With plasma spraying and electrophoretic deposition, the most common problems are cracking and flaking of the coatings, which are obviously a non-wanted property concerning implantable devices intended to survive years inside the body. Furthermore, laser ablation and sol-gel techniques are expensive, whereas high temperatures are required for plasma spraying, laser ablation, and electrophoretic deposition [61,62,63].

Atomic layer deposition (ALD) is a method that has many beneficial properties in contrast to the other methods described above. For instance, ALD can be used for coating complex three-dimensional surfaces on a nanometer scale, and the coating thickness can be controlled in detail, leading to very uniform and conformal coatings [64,65].

We have previously described the preparation of a nanocrystalline HA coating on Ti substrate by converting ALD-CaCO_3_ to HA by chemical treatment in a dilute phosphate solution [66]. We have also shown that the coating is intact [67] and suitable for cell culture, as osteoblast line cells attached to the coating, and the cell viability was normal [68]. Further, we have also successfully cultured human bone marrow-derived cells on the coating [66]. However, we have not previously characterized the foreign body reaction or osteoclast actions on the coating. To our knowledge, this is the first study of the cellular reactions occurring on the ALD-HA coating when monocytes come into contact with it. We hypothesized that a foreign body reaction follows, with the possible subsequent resorption of the coating. A resorbable coating could offer new insights for developing implant materials with improved osseointegration properties since most of the bone remodeling takes place at previously resorbed surfaces [22]. Previous studies have utilized different methods in producing the HA coating, but the coating produced by the ALD method utilized in this study has not been characterized before in regard to monocyte differentiation and resorbability. This is the first study of ALD-HA coating and primary human monocyte culture with 14 days culture period, which is considerably longer than the previously studied initial cellular reactions performed mostly with cell lines.

## 2. Results

### 2.1. Contact Angle and Surface Roughness Measurements

**Figure 1:**
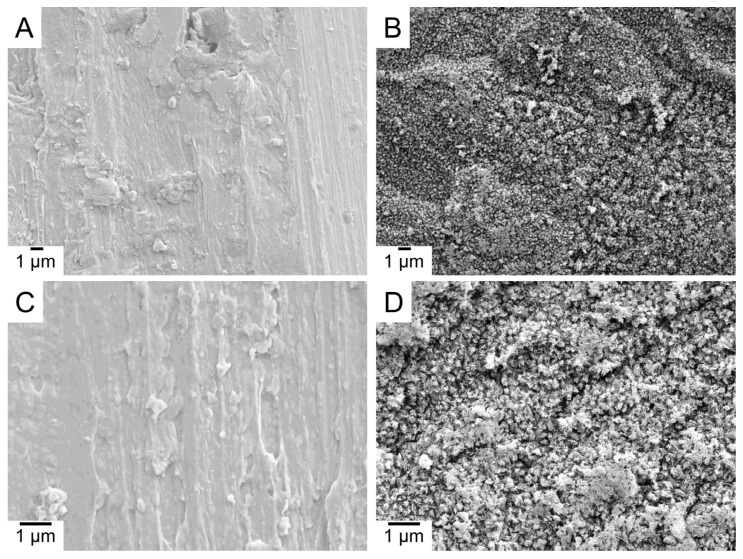
FESEM images of non-coated Ti sample (**A**,**C**) and ALD-HA (**B**,**D**). HA crystals appear on ALD-HA, whereas the non-coated Ti sample is devoid of them. Magnification 4000× (**A**,**B**) and 10,000× (**C**,**D**).

**Figure 2:**
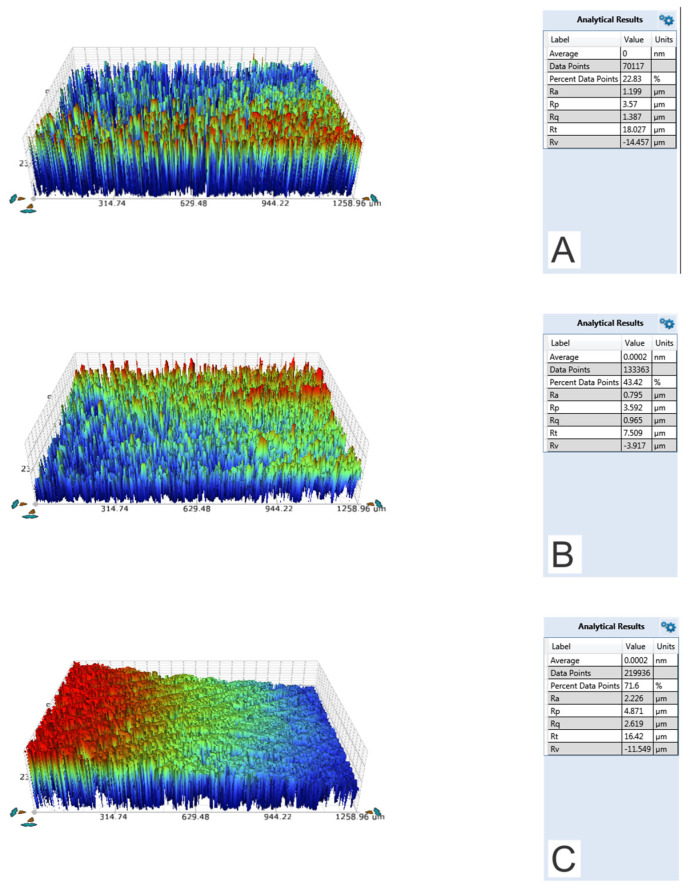
Typical 3D surface profile of non-coated Ti, ALD-HA, and bovine bone slice samples. The surface profiles of non-coated Ti sample (**A**) and ALD-HA surface (**B**) demonstrated a lower surface roughness value compared to the bovine bone slice profile (**C**). Images were captured using a 5× objective lens and a 0.5 multiplier.

The contact angle and surface roughness (Ra) values are shown in Table 1. The non-coated Ti sample, ALD-HA, and bovine bone slice showed similar hydrophilic contact angle values. However, the ALD-HA surfaces demonstrated significantly lower surface roughness (Ra 0.713 µm) compared to the bovine bone slice (Ra 2.30 µm; *p* < 0.001). The surface roughness of the non-coated Ti sample was 1.21 µm, which is between the values of ALD-HA and bone and differs from both (*p* < 0.001). The ALD-HA coating, therefore, smoothens the surface of the Ti sample. Figure 1 displays the surface of the non-coated Ti sample and ALD-HA. HA crystals can be seen on ALD-HA, whereas the non-coated sample is devoid of crystals. Figure 2 displays the 3D surface profiles of non-coated Ti sample, ALD-HA, and bovine bone slices. Note that the 0.713 µm roughness is much more than the HA film thickness (380 nm).

### 2.2. Multinuclear Foreign Body Cells Generated on ALD-HA

**Figure 3:**
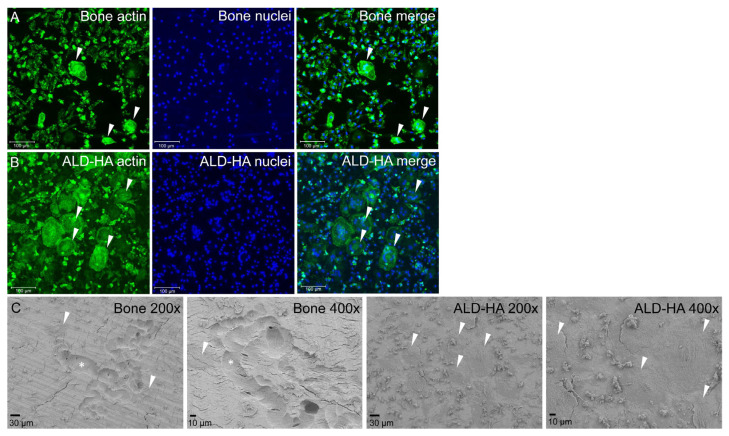
Confocal microscope (**A**,**B**) and FESEM (**C**) images of multinuclear cells and resorption on bone and ALD-HA. When stimulated with osteoclastogenic growth factors RANKL and M-CSF, peripheral blood monocytes differentiated into small multinuclear cells (arrowheads) with actin rings on bone (**A**), whereas on ALD-HA large multinuclear cells were generated (**B**). Bone resorption was evident on bone surface ((**C**); asterisks), and occasional small osteoclasts (arrowheads) were observed in or near the resorption pits. ALD-HA was devoid of resorption pits but was covered with large, flat multinuclear cells (arrowheads).

We have previously studied osteoclast formation from peripheral blood monocytes on bone [69]. During the 14 days culture, stimulated with the growth factors RANKL and M-CSF, the monocytes undergo differentiation into multinuclear bone-resorbing osteoclasts. This setup was used as a control in this study. The multinuclear cells on the bone surface with actin rings are shown in Figure 3A, and the resorption pits imaged with FESEM are shown in Figure 3C. When monocytes were cultured on ALD-HA with the osteoclastogenic growth factors, large multinuclear cells were observed in the samples (Figure 3B). The actin rings in multinuclear cells on bone were bright and thick, whereas, on ALD-HA, the rings were paler and more diffuse. Our previous study [69] shows that the average nuclei number in human osteoclasts differentiated from peripheral blood monocytes is 4. In this study, we did not analyze the nuclei number due to the limited number of samples. Still, the confocal microscopy images show that the nuclei number of multinuclear cells on the ALD-HA surface is substantially larger than on bone. In addition, the FESEM images show the large flat multinuclear cells covering the ALD-HA surfaces uniformly, whereas, on bone, only occasional, smaller osteoclasts are observed in or near the resorption pits. The total lack of resorption pits on ALD-HA suggests that the multinuclear cells on ALD-HA are foreign body cells instead of osteoclasts.

## 3. Discussion

We have previously demonstrated osteoblast lineage cell adhesion on the ALD-HA surface with focal adhesion-like structures [68]. The purpose of this work was to continue cell adhesion studies with monocytes and to investigate whether osteoclastogenesis or foreign body reaction follows the cell attachment. To our knowledge, these important cellular reactions have not been previously studied on ALD-HA. Our results show that peripheral blood monocytes undergo differentiation into bone-resorbing osteoclasts on the bone surface, but on the ALD-HA coating, the monocytes differentiate into foreign body cells without the capacity to resorb the coating. Similar results were obtained in our previous study, where human bone marrow mononuclear cells were cultured on bone and ALD-HA and differentiated into multinuclear cells [66]. Other studies have also observed the large, flat morphology of the multinuclear cells cultured on HA [45,52]. Comparable morphology was also present in studies where large multinuclear cells appeared on nacre [70] or HA [46,71], compared to smaller osteoclasts on bone. Chappard et al. [70] described the properties of different types of giant cells and suggested that a certain type of giant cell is induced on biomaterials. This giant cell has a nonuniform distribution of nuclei, and their differentiation is not associated with inflammation. In addition to HA and nacre, various other biomaterials have been shown to induce different types of giant cells. Their morphology and characteristics were reviewed by Al-Maawi et al. [72]. In our samples, the multinuclear cells on ALD-HA had a fairly central location of the nuclei, but also a few Touton cell-like (crown-resembling central organization of nuclei) and Langhans cell-like (horseshoe-resembling organization of nuclei) giant cells were observed. Therefore, it is possible that several different types of giant cells are induced on our ALD-HA coating.

Considering biomaterial degradation by multinuclear cells, Ghanaati et al. [73] demonstrated faster degradation of beta-tricalcium phosphate (β-TCP) granules compared to HA granules implanted into goat muscle tissue. In their study, HA granules remained intact in the implanted tissue for a longer time compared to β-TCP, but eventually, HA granules started to show dissolution by multinuclear cells. In addition, the multinuclear cells induced with β-TCP presented a more intense TRACP-staining, probably depicting their increased degradation ability compared to cells on HA granules. Although the multinuclear cell types in their study were not specifically identified as foreign body cells or osteoclasts, it is possible that β-TCP induced resorptive osteoclasts, but HA induced foreign body cells with limited dissolution capacity. This would be in line with our results concerning the multinuclear cells on bone and HA. In addition, the slow degradation process of implanted HA materials has also been noted by Okuda et al. [74] Therefore, it is possible that our 14 days cell culture period was sufficient to induce the initial foreign body reaction, but not the possible subsequent osteoclastogenesis and resorption of the material, as described by Sabokbar et al. [44]. Furthermore, we could not detect any dissolution of the ALD-HA surface by the multinuclear cells, contrary to a study by ten Harkel et al. [32], which demonstrated that although not capable of bone resorption, the FBGCs dissolved HA-like calcium phosphate coating. However, even when human peripheral blood monocytes were differentiated into osteoclasts and cultured for 21 days by Ciapetti et al. [54], no resorption pits were observed on biomimetic HA coatings. In contrast, resorption was evident on stoichiometric sintered HA, which is additional proof of the major effects the surface topography has on osteoclast fusion and activity, also observed in other studies [53,54,55,75,76,77,78,79].

Surface wettability can be estimated by measuring the CA, which represents the angle between the tangent line to a drop surface of the liquid at the three-phase boundary and the horizontal surface of the solid interface. Generally, the water CA ranges from 0 to 180° and is considered hydrophilic at a value lower than 90° and hydrophobic when the value is more than 90°. CA values close to 0° are regarded as superhydrophilic, whereas above 150 are regarded as superhydrophobic [80]. In our study, the CA values were 86.9 (3.2), 86.2 (4.5), and 86.7 (4.5) for the non-coated Ti sample, ALD-HA, and bovine bone slice, respectively, indicating slightly hydrophilic surfaces for all investigated materials. Surface chemistry plays an essential role in determining wettability, as indicated in a study by Surmeneva et al. [81]. Their study showed that the deposition of a nano-HA film onto the titanium surface led to a decrease in the water contact angle from 130 ± 5° to 50 ± 5°. Additionally, the surface topography and roughness can affect wettability. However, similar wettability behavior was observed for the ALD-HA and bovine bone slice surfaces despite the significant difference in roughness values of (0.713 µm) and (2.30 µm), respectively. ALD-HA coating of the titanium surface used in this study decreased the surface roughness from 1.21 (±0.05) to 0.713 (±0.08) µm.

Studies have shown that the implant surface topography and chemical composition impact osteoclast activity [53,54,55,75,76,77,78,79]. In general, rough surfaces promote increased osteoclastogenesis compared to smooth surfaces [75,76,77,78]. The same applies to calcium phosphate coatings as well. However, considering osteoclastogenesis and osteoclast activity, there seems to be a window for the optimal surface architecture since submicron-scale (≤1 µm) coatings were found more favorable compared to micron-size (≥1 µm) [50,52,82,83,84] or nanoscale (1–100 nm) coatings [49,51,85]. This could explain the contradictory results about the capacity of osteoclasts to resorb the HA coating [18,19,21,25,32,45,47,48]. Considering our results, it is possible that some of the multinuclear cells on ALD-HA are osteoclasts, but the surface topography of our samples does not support resorption, although the Ra value of ALD-HA (0.713 µm) is in the optimal range. In fact, the actin rings we observed in the cells on ALD-HA very much resembled the ones that Costa et al. [52] described as the characteristics of non-resorbing cells since the rings are more diffuse and paler compared to the thick and bright structures observed in osteoclasts on bone. It seems that the rougher surface of the bone (Ra 2.300 µm) supports osteoclastogenesis and resorption, but the topography of the ALD-HA surface does not induce the same reaction from peripheral blood monocytes in our study. In addition to the topographical and chemical differences between the bone and ALD-HA surfaces, it is of note that several other factors, such as pH and the ions released from the surface can have a major impact on the cellular reactions, as reviewed recently [86]. This especially concerns osteoclasts, as they are also in vivo regulated by H^+^, Ca^2+^, and PO_4_^3−^ ions, which are the components of both bone and HA and might be released from these materials in different amounts [54].

Although the foreign body reaction, including the differentiation of multinuclear FBGs and osteoclasts, is often thought to be the unwanted process leading to implant loosening, different perspectives consider these events natural and crucial for osseointegration. Trindade et al. [87] discussed these events in a recent review and expressed an opinion of osseointegration being a consequence of the foreign body reaction, upon the condition that the cellular responses are tightly controlled and in equilibrium, without excess differentiation of multinuclear cells and the following osteolysis. Furthermore, considering ALD coatings, Smieszek et al. [88] observed increased viability and activity of osteoblast lineage MC3T3 cells on ALD TiO_2_ coating, but osteoclast activity on the coating was decreased. We obtained previously similar results with MC3T3 osteoblasts on ALD-HA [68]. It is possible that the observation of Smieszek et al. on the inhibited preosteoclast invasion on the ALD TiO_2_ coating could also explain our results in this present study. Although the observation in the study by Smieszek et al. considered a co-culture of MC3T3 osteoblasts and preosteoclasts, it is of note that peripheral blood contains small amounts of mesenchymal stem/stromal cells [89,90], which might behave in a similar way to osteoblast lineage cells on the ALD coating, i.e., prevent the attachment of osteoclast lineage cells. The mesenchymal stromal cells are osteoblast precursors, which regulate osteoclastogenesis [91,92] and could be the mediators behind the effects observed in this study. If these cells attach firmly to the smoother ALD coating, they prevent preosteoclast attachment, and further, their altered metabolic activity favors FBGC differentiation instead of osteoclasts. As Smieszek et al. discussed, this is beneficial for osseointegration since excess osteoclast activity is inhibited. However, these explanations concerning this study are hypothetical and require further investigation, but nevertheless offer an interesting approach for studying the initial cellular reactions on ALD-produced coatings, which, at present, are mainly unknown. Moreover, as Ciapetti et al. [54] and He et al. [55] noted, the optimal biomaterial surface should support all types of cells in contact with it, such as osteoblasts, osteoclasts, and macrophages, but the properties of different materials might not be beneficial for all these cells at the same time. Therefore, further studies, including co-cultures with all types of bone cells, are required, especially as recent studies have focused mainly on one cell type at a time, which does not represent the complex situation in vivo.

## 4. Materials and Methods

### 4.1. Preparation of Nanocrystalline HA Coating (ALD-HA) on Ti Substrates with ALD Method

The HA coatings were fabricated on Ti substrates as described by Avila et al. and Holopainen et al. [66,67]. The substrate for ALD-HA coating was a 5 cm × 5 cm (25 cm^2^), 1 mm thick titanium sheet (Grade 2, ASTM B265 specification, William Gregor Ltd., London, UK). The ALD coating was started by depositing a thin film of CaCO_3_ in an F-120 ALD reactor (ASM Microchemistry Ltd., Helsinki, Finland) with nitrogen carrier and purging gas. The CaCO_3_ films were deposited using the Ca(thd)_2_—O_3_ process previously reported in the literature [93]. Ca(thd)_2_ (Volatec Oy, Vantaa, Finland) was evaporated at 188 °C, and O_3_ was generated from O_2_ (99.9999%) with a Wedeco Ozomatic Modular 4 HC Lab ozone generator. Samples were produced with 4000 ALD cycles resulting in a thickness of 380 nm. Pulses and purges of 1 s were used for all precursors. The depositions were conducted at 250 °C. Conversion of CaCO_3_ to HA was achieved by using 0.2 M (NH_4_)_2_HPO_4_ (99%; Merck KGaA, Darmstadt, Germany) solution at 95 °C. After conversion, the samples were rinsed with deionized water and blown dry with compressed air. A manual plate cutter (Bernardo PTS 1050 S Manual disc cutter, Linz, Austria) was used for cutting the ALD-coated titanium plates. The Ti plate was firmly placed in a disc pressing to keep it in place during the cutting process. The plate was cut to produce 1 cm^2^ square-shaped size discs. Before cell culture, the samples were soaked in 70% ethanol for 10 min and air-dried.

### 4.2. Contact Angle and Surface Roughness Measurements

The surface contact angle (CA), which represents surface wettability, of ALD-HA and bovine bone slice were evaluated using the sessile drop method described by Jong et al. [94]. An Optical tensiometer (Theta, Biolin Scientific Oy, Espoo, Finland) was used for water CA measurements. Five randomly selected points per sample were evaluated. For each droplet, 120 images were recorded in 20 s. The Young–Laplace equation was used to define the CA around the droplet. The surface roughness averages (Ra) of ALD-HA and the bovine bone specimens were measured using a 3D non-contact optical profilometer (Bruker Nano GmbH, Billerica, MA, USA). A total of 5 randomly selected points per sample were evaluated with a 5× objective lens and a 0.5 multiplier, using a back scan and length parameters of 20 μm and 60 μm in VSI/VXI mode to obtain a 3D image of the specimen surfaces. Software (Vision 64) was used to generate surface areas and roughness parameters.

### 4.3. Monocyte Differentiation on Bone and ALD-HA

The isolation and culture protocol for peripheral blood monocytes were modified from [95]. The cells were collected from whole blood samples from 3 healthy female donors aged between 39–45 years, who provided informed consent. The study was approved by the Ethical Committee of The Northern Ostrobothnia Hospital District (Oulu University Hospital EETTMK: 180 and 191/2001; 12/2004; 35/2009; 29/2011). Blood was diluted 1:1 in PBS and layered over Ficoll-Paque Premium solution (GE Healthcare, Little Chalfont, UK). The samples were centrifuged at 400× *g* for 35 min following the manufacturer’s protocol. Mononuclear cell layer was collected and centrifuged twice at 190× *g* for 10 min in PBS and finally suspended in α-MEM (Corning Life Sciences) containing 10% FBS (Biowest, Riverside, MO, USA), 100 IU/mL penicillin, and 100 µg/mL streptomycin (Sigma-Aldrich, St. Louis, MO, USA). A total of 300 000 cells (9.4 × 10^5^ cells/cm^2^) were layered on sonicated bovine cortical bone slices (0.28 cm^2^; Lehenkari Consulting, Oulu, Finland) in 96-well plates (Cellstar; Greiner Bio-One, Kremsmünster, Austria) in a final volume of 200 µL. Then, 2 × 10^6^ cells (1.05 × 10^6^ cells/cm^2^) were layered on ALD-HA samples (1 cm^2^) in 24-well plates (Cellstar; Greiner Bio-One) in a final volume of 1 mL. Osteoclastogenesis was induced with 20 ng/mL RANKL (PeproTech EC, London, UK) and 10 ng/mL M-CSF (R&D Systems, Minneapolis, MN, USA). Half of the media was changed every 3–4 days (100 µL/well for bone slices and 500 µL/well for ALD-HA). At 1-week time point, all media was changed (200 μL/well for bone slices and 1 mL/well for ALD-HA). Cells were cultured at +37 °C (5% CO_2_, 95% air) for 14 days.

### 4.4. Confocal Microscopy

The cells were fixed with 4% paraformaldehyde (PFA) in PBS for 10 min. The actin cytoskeleton was stained with Alexa 488-conjugated phalloidin (200 U/mL stock diluted 1:100 in PBS; Invitrogen Europe, Paisley, UK) for 20 min at +37 °C. Nuclei were stained with Hoechst 33258 (1 mg/mL stock diluted 1:800 in PBS; Sigma-Aldrich) for 10 min at RT. The samples were mounted in 70% glycerol-PBS. The cells were viewed with Leica TCS SP8 confocal with a DMI8 microscope using LAS X 3.5.2 acquisition software. The used objective was an HC PL APO CS2 CS2 20 × 0.75 DRY. Samples were imaged with 488 nm and 405 nm solid-state lasers; the pinhole was set to Airy 1 and scan speed to 600 Hz. Maximum intensity projections (MIP) were created from the Z-stacks.

### 4.5. Field Emission Scanning Electron Microscopy (FESEM)

The bone slices, non-coated Ti samples, and ALD-HA samples were dehydrated in ascending ethanol series and dried with a critical point drying equipment K850 (Quorum Technologies, Lewes, UK). Samples were coated with 5 nm platinum by Q150T ES sputter coater (Quorum Technologies, Lewes, UK) and viewed with Sigma HD VP FE-SEM (Carl Zeiss Microscopy GmbH, Oberkochen, Germany). FESEM images were taken with 5.0 kV voltage.

## 5. Conclusions

We demonstrated for the first time that human peripheral blood monocyte differentiation on bone and ALD-HA leads to different outcomes, as on bone osteoclastogenesis is induced, but on ALD-HA, a foreign body reaction is observed. On bone slices, the small multinuclear cells were able to resorb the substrate generating typical osteoclastic resorption pits. In contrast, on ALD-HA, the multinuclear cells were remarkably large with several nuclei but lacked the resorption capacity of the surface. We did not detect differences in the surface wettability between bone and ALD-HA, and both surfaces were found to be hydrophilic. The surface roughness, however, was found to be greater on bone, which might support osteoclastogenesis, compared to the smoother surface in the ALD-HA samples. These results provide knowledge about the initial cellular reactions of ALD-HA, which is of great importance when developing new types of implant surfaces with modern methods. With the modifications of this technique, new types of nanothin coatings can be produced for orthopedic and dental implants with conformal and porous surfaces.

## Figures and Tables

**Table 1:** Mean and standard deviation of water contact angle and surface roughness determination on non-coated Ti sample, ALD-HA, and bovine bone slice. ^a^ *p* < 0.001, non-coated Ti sample vs. ALD-HA or bone; ^b^ *p* < 0.001, ALD-HA vs. bone.

	Contact Angle (°), Mean (SD)	Surface Roughness (Ra; µm), Mean (SD)
Non-coated Ti sample	86.9 (3.2)	1.21 ^a^ (0.05)
ALD-HA	86.2 (4.5)	0.713 ^b^ (0.08)
Bone	86.7 (4.5)	2.30 (0.18)

## Data Availability

The data presented in this study are available in the article.
